# Evaluating the First CE‐Marked LLM‐Based Chatbot for Evidence‐Based Neurology

**DOI:** 10.1111/ene.70736

**Published:** 2026-08-18

**Authors:** Patricia Kirschner, Paula Z. Epping, Sven G. Meuth, Marc Pawlitzki, Lars Masanneck

**Affiliations:** ^1^ Department of Neurology, Medical Faculty and University Hospital Düsseldorf Heinrich Heine University Düsseldorf Düsseldorf Germany

**Keywords:** AI, digital health, evidence‐based medicine, large language models, neurology

## Abstract

**Background:**

Large language models (LLMs) are entering clinical workflows for information retrieval and decision support, but concerns persist about factual accuracy and traceability to evidence. Prof. Valmed is the first CE‐marked LLM‐based tool for medical information retrieval in Europe, raising the question whether certification aligns with reliable performance in neurology. This study aims to assess the answer accuracy of Prof. Valmed on a neurology benchmark and compare its performance with previously evaluated commercial LLM tools.

**Methods:**

Prof. Valmed (V2.0.1_2.0.0) was tested on a 130‐item benchmark derived from American Academy of Neurology guidelines (65 case‐based, 65 knowledge‐based). Each question was prompted four times. Two raters scored responses as correct, inaccurate, wrong, or refused; disagreements were adjudicated. Modal ratings per question were used for analysis. Comparative performance against 16 LLMs or configurations previously assessed using the same protocol was evaluated.

**Results:**

Prof. Valmed achieved 76.2% correct answers, 14.6% inaccurate, 6.9% wrong, and 2.3% refused. In pairwise comparisons, Prof. Valmed performed significantly better than 8 models, comparable to several retrieval‐ or whitelist‐enabled tools, and significantly worse than one reasoning model with whitelisting of neurology sources. Overall, its performance was within the range of standard commercial tools.

**Conclusion:**

The CE‐marked product demonstrated accuracy comparable to contemporary LLMs but did not outperform reasoning‐enabled systems. CE marking ensures regulatory conformity rather than inherent performance advantage, and the certification process may limit the integration of rapidly evolving model architectures into approved systems. Future evaluations should examine reasoning quality, source use, and update feasibility across medical domains.

## Graphical abstract

Prof. Valmed, the first CE‐marked LLM‐based medical retrieval tool in Europe, was tested and compared using an established neurology benchmark. Its performance was comparable to most other evaluated tools but did not outperform a reasoning‐enabled system using whitelisted neurology sources. Certification of LLMs as medical devices supports regulatory certainty, but benchmark validation remains essential to demonstrate clinical performance.

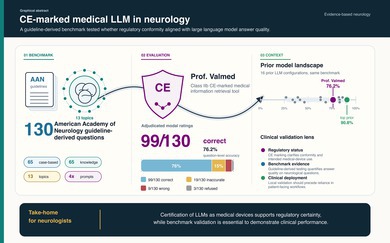

**Abbreviations**
AAN: American Academy of Neurology
AI: Artificial intelligence
LLM: Large language model
RAG: Retrieval‐augmented generation
WL: Whitelisting
*κ*
: Cohen's kappa

## 1 Introduction

The use of large language models (LLMs) has grown rapidly in various professional fields and such models are increasingly adopted by healthcare professionals [1]. These systems can not only be used to extract and interpret clinical data but also have the potential to streamline workflows and support clinical decision‐making [2, 3]. In a field where factual accuracy and the use of evidence‐based information sources are integral to upholding patients' safety, the clinical use of LLMs presents challenges. Responses of LLMs are frequently prone to errors in factual accuracy and can exhibit “hallucinations”, in which the model generates answers and citations that seem fluent and convincing but are entirely false or fabricated [4]. By utilizing retrieval augmented generation (RAG), a technique in which LLMs are provided with vetted external information sources (e.g., clinical guidelines or peer‐reviewed scientific articles), these phenomena can be reduced and a model's answer quality optimized [5, 6]. To “ground” the models' answers in factual context, more and more commercial LLM providers further offer an integration of web‐search into LLM answers, which also leads to improvements in a medical context [7]. By “whitelisting”, in which an LLM's information access is restricted to a predefined set of verified and approved (online) sources, answer quality and source traceability can be enhanced during such artificial intelligence (AI)‐assisted web‐search [7].

Since 2023, a growing number of studies, most of them testing general‐domain tools rather than models specialized for medical use, have evaluated the use of LLMs in clinical medicine, including in emergency medicine and neurological settings [5, 8, 9, 10, 11, 12, 13]. However, given the rapid evolution of LLMs and increasing availability of medical‐specific programs, up‐to‐date data on the accuracy and practical value of new AI tools are needed. In this context, efforts are underway to integrate LLMs into medical products or, through structured quality assurance and auditability processes, enable CE certification of the digital tools themselves as medical devices. As very recently the first CE‐marked LLM‐based tool for information retrieval has been released in Europe, this discussion and the need for information on LLM performance and liability has further gained ground.

Prof. Valmed is the first CE‐marked LLM‐based product that offers medical information retrieval to answer diagnostic and therapeutic questions [14]. The subscription‐based tool, which is available in both English and German, is based on an undisclosed LLM model and, according to the manufacturer, uses a curated proprietary medical database [15]. The tool's reliability and safety were evaluated according to the requirements of the European Medical Device Regulation and it is now marked as a medical device in the risk class IIb, classifying it as medium to high risk device [14]. However, the methods and results of the tests resulting in the certification are not publicly available at the time of writing this article. So far, scientific reports evaluating the tool's accuracy in answering clinical questions are limited to a rheumatological study assessing diagnostic accuracy and a study comparing the tool with other LLMs and students on multiple sclerosis exam questions [16, 17]. Diagnostic performance was comparable, albeit slightly inferior, to OpenEvidence and a GPT‐5 LLM in the rheumatology study [17]; in contrast, on the multiple sclerosis dataset it was nominally higher than the comparators on the MS exam dataset, though the difference was not statistically significant [16].

The CE marking of an LLM‐based medical product is intended to provide greater regulatory transparency and assurance for professional medical use; however, the comparative performance of such medically regulated LLMs relative to commercially available general‐domain LLMs has not yet been systematically evaluated. This study aims to test the accuracy of the answers provided by Prof. Valmed to questions concerning a broad range of neurological diseases and compare the results with those reported for general‐domain LLM tools in two previous studies [7, 18]. In those prior publications, various LLMs with and without RAG were evaluated using a 130‐question benchmark based on guideline recommendations by the American Academy of Neurology (AAN) [7, 18]. In this study, Prof. Valmed was tested using the same benchmark to evaluate the answer accuracy of this newly CE‐marked LLM.

## 2 Methods

### 2.1 Labeling Setup and Dataset

We evaluated the performance of the LLM‐based chatbot Prof. Valmed (Version V2.0.1_2.0.0) using a previously established 130‐item AAN benchmark, which comprises 65 clinical‐case questions and 65 factual‐knowledge questions [18].

Prof. Valmed is a generative AI platform certified as a Class IIb medical device under the European Union Medical Device Regulation [14]. It is designed to provide validated medical information to healthcare professionals. The model employs a proprietary database that integrates curated and validated medical sources such as guidelines, regulatory authority documents, medical society publications, and clinical studies. However, the exact mechanisms behind the model are not publicly known.

To ensure direct comparability with previous evaluations of other LLM‐based tools, the same benchmark dataset, containing 130 neurological questions, and rating protocol as in previous studies [7, 18] was used: The underlying items have been developed from AAN guidance and were partially paraphrased. They cover 13 neurological topics (see Table S1), and the full list of benchmark questions is available in a previous publication [18]. Half of them are clinical vignettes that require applying multiple recommendations to a scenario rather than matching page wording. Each question was prompted four independent times to capture answer variance (identical system). All prompts were executed between 3 and 24 June 2025 without any source filters. Prof. Valmed was accessed independently by the authors using a publicly available free trial version that included the full functionality of the tool. No collaboration or financial relationship with the company exists in the context of this study.

### 2.2 Labelling Process

Labelling was conducted as described previously independently by two raters, with a third rater resolving disagreements between the two primary raters [18]. Raters were instructed to mark an answer as “correct” if the recommendation itself was accurate, even if minor reasoning errors were present. Responses that were partially incomplete or contained minor errors that could lead to a misunderstanding of an otherwise generally correct answer were classified as “inaccurate”. Responses labeled “wrong” either failed to answer the question properly or contained incorrect, very incomplete, or potentially dangerous information. If the model refused to answer a question, it was rated as “refused”.

As Prof. Valmed reports the sources that its answers are based on, we further marked those answers that included AAN sources. Because the tool did not show meaningful “source hallucination”, a hallucination sub‐analysis was not conducted.

### 2.3 Statistical Analysis

To compare Prof. Valmed's performance against other models, we used modal ratings aggregated by question number (compatible with previous work). Cohen's kappa (*k*) was calculated between the two primary raters. Pairwise Wilcoxon signed‐rank tests compared Prof. Valmed to each other model, with Holm‐Bonferroni corrections for multiple comparisons. Mean differences indicated the direction of performance differences. Questions for which Prof. Valmed refused to provide an answer were excluded from all quantitative and pairwise statistical analyses to maintain comparability across models. No formal prospective power calculation was performed because the analysis used the complete previously established benchmark rather than a sampled subset. The primary unit of analysis was the benchmark question.

We conducted two ordinal logistic regression analyses to assess factors influencing answer quality. Ratings were encoded ordinally (Correct > Inaccurate > Wrong); refused answers were excluded from regression analyses. For each analysis, we fitted an ordered logit model. The first analysis tested the effect of AAN citation presence using a single binary predictor. The second analysis tested question type (knowledge vs. case) using a binary predictor. *p*‐values are reported for regression coefficients, with odds ratios and 95% confidence intervals indicating the magnitude of effects. Effect sizes were calculated as mean paired differences in modal ordinal rating scores between Prof. Valmed and each comparator model. Ratings were encoded as correct = 2, inaccurate = 1, and wrong = 0. The difference was calculated as Prof. Valmed minus comparator for each matched question and then averaged across all analyzable paired questions. Analyses were done in Python 3.9.6 using the packages pandas (2.2.3), numpy (2.0.2), scipy (1.13.1), statsmodels (0.14.4) and matplotlib (3.9.4).

### 2.4 Ethical Considerations

No human subjects were involved in this research. All analyses are based on a previously published dataset without any actual patient data. An Institutional Review Board vote could thus be waived.

## 3 Results

**FIGURE 1:**
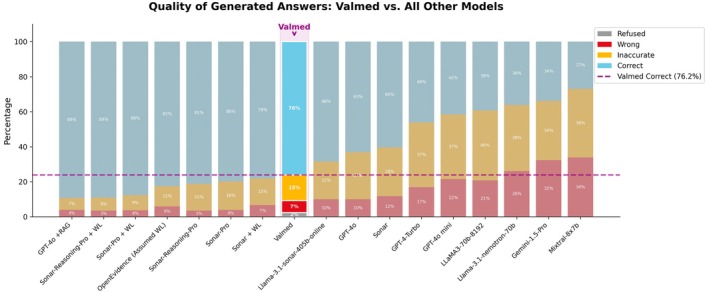
Response quality of Prof. Valmed in comparison to other LLM models with different configurations. Quality of generated answers of Prof. Valmed in comparison to other LLMs with different system configurations (with and without RAG, with and without whitelisting of AAN domains). Performance results for all models other than Prof. Valmed have been reported previously [7, 18]. The exact technical setup of OpenEvidence is unknown, but some sort of whitelisting or indexing is assumed (Assumed WL). All models were evaluated on the same 130 benchmark questions. For Prof. Valmed, four repeated responses per question were aggregated by modal question‐level rating; 3/130 questions were refused. Pairwise statistical comparisons used 127 matched question‐level observations after exclusion of these refused questions. Bars show the percentage of benchmark questions judged “Correct”, “Inaccurate”, “Wrong”, or “Refused”; comparator bars are intentionally desaturated, while Prof. Valmed is shown in full color. A pairwise comparison on the modal values can be found in Table S3. AAN, American Academy of Neurology; RAG, retrieval augmented generation, WL, Whitelisting.

Prof. Valmed generated a total of 520 answers to 130 different questions. Inter‐rater agreement between primary raters of answer quality was excellent at *κ* = 0.95. To these questions, 76.2% of answers produced by Prof. Valmed were correct (see Figure 1). The raters evaluated 14.6% of answers as inaccurate and 6.9% as wrong. Three questions (one knowledge‐based and two case‐based questions on brain death diagnostics), equaling 2.3% of the total answers, were refused by Prof. Valmed and could thus not be evaluated qualitatively by the raters (see Table S2). Rating variability between the answers of Prof. Valmed to the four repetitions of each question was low (13.1%, 17/130 questions).

**FIGURE 2:**
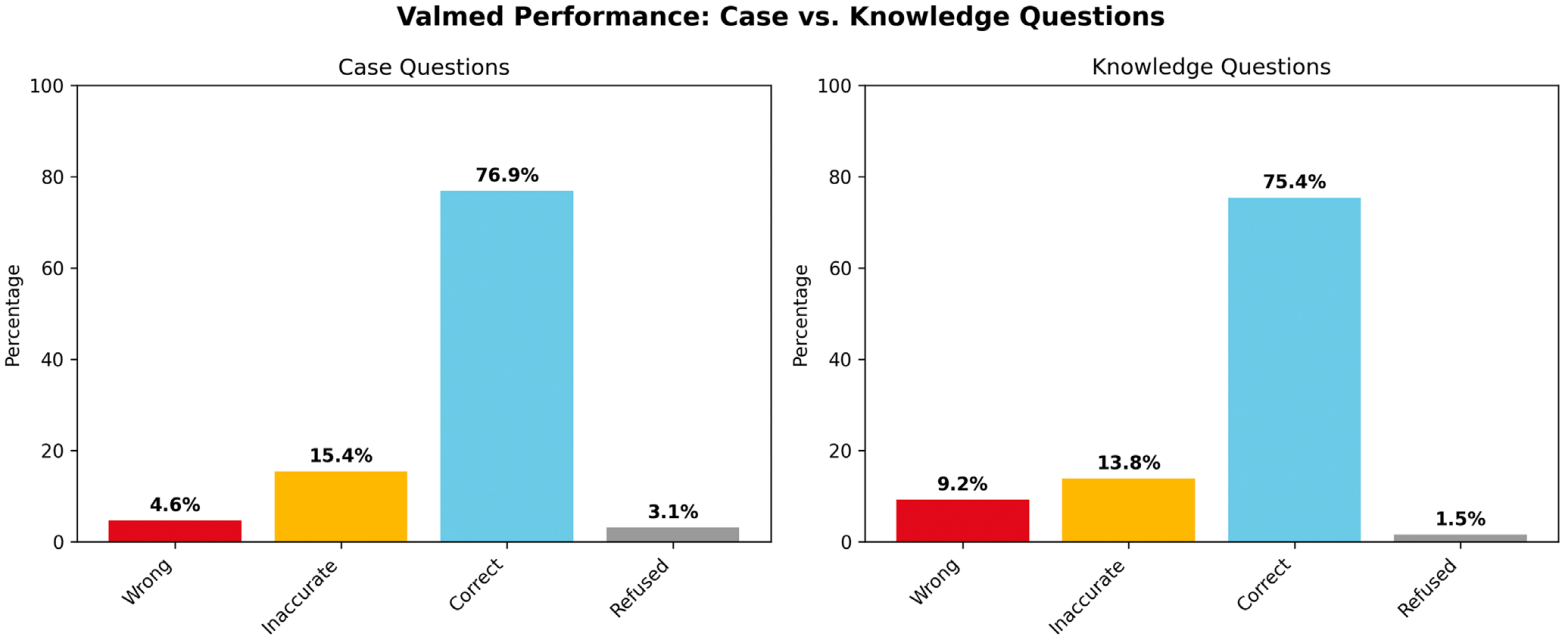
Response quality of Prof. Valmed to case‐based versus knowledge‐based questions Subanalysis of the quality of answers generated by Prof. Valmed to case‐based (left) and knowledge‐based (right) questions. The 130 questions used in this study covered 13 neurological topics, for which recent AAN‐guidelines are available. From each guideline, five relevant clinical aspects were derived by two neurologists and one case‐based and one knowledge‐based question were created for each of the selected clinical aspects. Bars show the percentage of responses judged “Correct” (light blue), “Inaccurate” (orange) or “Wrong” (red) as well as the percentage of answers refused by Prof. Valmed for each category. AAN, American Academy of Neurology.

Citation of an AAN guideline in the answer provided by Prof. Valmed showed a non‐significant trend toward higher response quality (OR = 1.6525; *p* = 0.282; 95% CI = 0.66–4.12). We also analyzed the influence of the question type on answer quality. As visualized in Figure 2, Prof. Valmed performed similarly on case‐based (76.9% correct answers) and knowledge‐based questions (75.4% correct answers). No statistically significant difference in response quality was observed between knowledge‐based and case‐based questions (OR = 0.81; *p* = 0.625).

**FIGURE 3:**
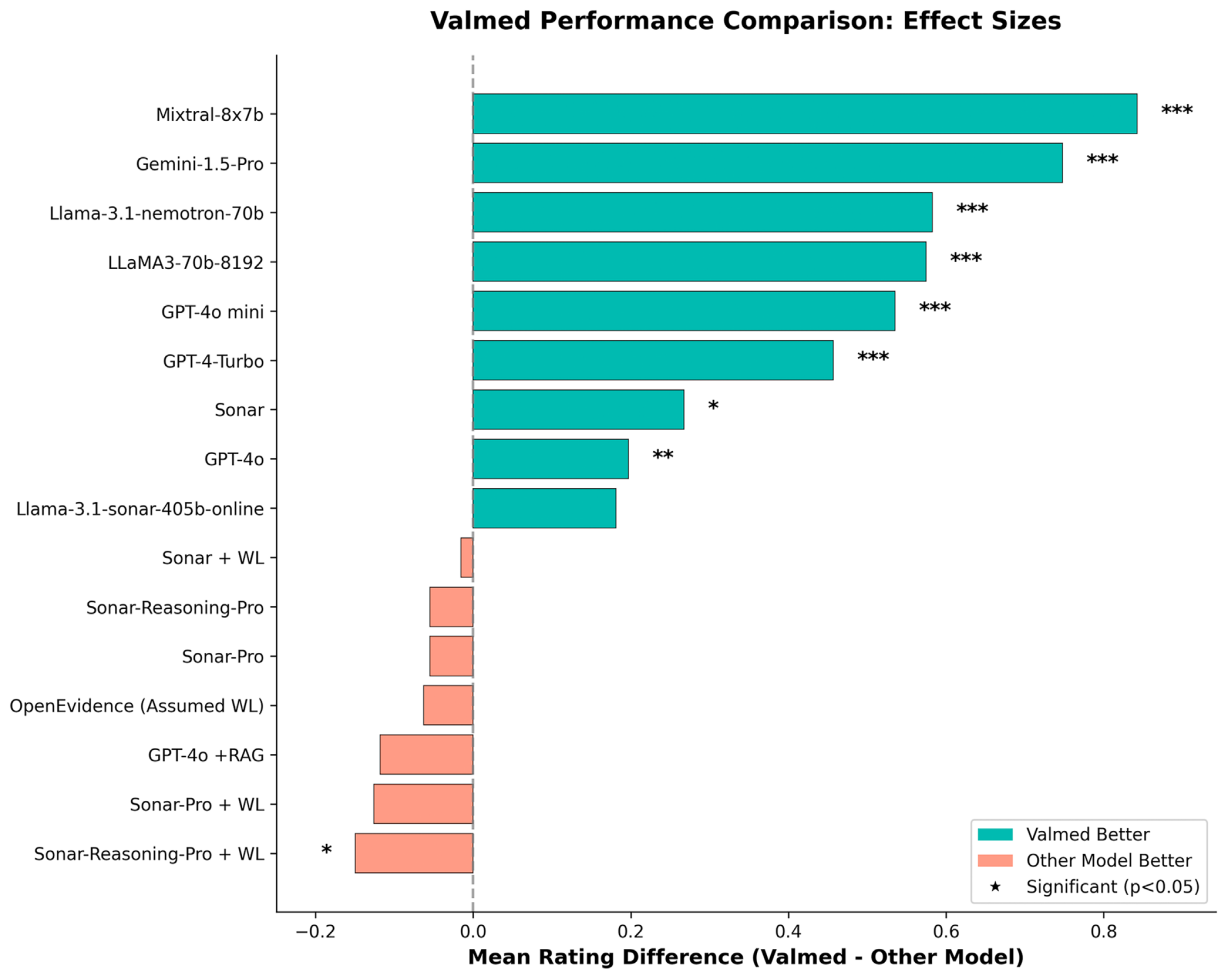
Effect sizes of the comparison of the response quality of Prof. Valmed to other LLMs Visualization of the mean rating difference between Prof. Valmed and other LLMs with different configurations (with or without RAG, with or without whitelisting of aan.com and neurology.org for web‐search‐enabled Perplexity models). The performance of Prof. Valmed was compared with previously published results from other models [7, 18]. As the exact technical setup of OpenEvidence is unknown but results are indicative of these configurations, whitelisting or indexing is assumed for this model (Assumed WL). Mean differences favored Prof. Valmed in nine comparisons with LLM configurations, eight of which were significant (green), whereas they favored the comparator in six comparisons (red). Significance levels are indicated. Effect sizes are mean paired differences in modal ordinal rating scores, calculated as Prof. Valmed minus comparator model. Ratings were encoded as correct = 2, inaccurate = 1, and wrong = 0. Questions refused by Prof. Valmed were excluded, yielding 127 paired questions per comparison. Positive values favor Prof. Valmed; negative values favor the comparator. Statistical significance was assessed using paired Wilcoxon signed‐rank tests with Holm correction for multiple comparisons. RAG, retrieval augmented generation; WL, whitelisting.

In a pairwise comparison with 16 other LLMs utilizing pairwise Holm‐adjusted Wilcoxon tests, Prof. Valmed achieved significantly better results than eight of them. Only one model (Sonar‐Reasoning‐Pro with whitelisting of aan.com and neurology.org) performed significantly better than Prof. Valmed (*p* = 0.006). Mean paired differences (Δ) on the 0–2 scale were −0.15 (adj. *p* < 0.048) for Sonar Reasoning‐Pro with whitelisting. Prof. Valmed was further outperformed by six other models, though these differences did not reach statistical significance. On the other hand, the CE‐marked tool performed significantly better than all previously tested models without RAG or online‐search, including the basic Perplexity Sonar model with web search capabilities. When comparing with other RAG‐ and web‐search‐enabled models it performed approximately on par with non‐reasoning whitelisted models (e.g., the basic Sonar Perplexity model reduced to searching aan.com and neurology.org) (see Figure 3 and Table S3).

## 4 Discussion

### 4.1 Comparative Performance of Prof. Valmed

In this comparative analysis, the CE‐marked, medical LLM‐enabled tool Prof. Valmed demonstrated comparable accuracy to the majority of models previously tested with the same benchmark. It achieved significantly better results than eight previously evaluated commercially available models, all of which had been assessed without whitelisting or RAG. However, one large‐scale reasoning model that presented with the highest answer accuracy in our previous analysis [7] (Sonar‐Reasoning‐Pro with whitelisting of two neurological websites) demonstrated significantly higher accuracy, while six additional LLM settings numerically outperformed Prof. Valmed, although these differences did not reach statistical significance.

With over 75% of questions answered correctly, it reached similarly high results for case‐based as well as knowledge‐related questions. With these results, Prof. Valmed was similar in performance to many of the previously best performing models. Interestingly, other models had higher differences between the accuracy of answers to case‐based and knowledge‐related questions in prior analyses, where especially the simpler models performed better on knowledge‐related questions [7, 18]. In contrast to the other models tested, Prof. Valmed refused to answer several questions, all related to brain death diagnostics. As this behavior was specific to the CE‐marked tool, it may reflect intentionally implemented safety mechanisms or regulatory requirements associated with the CE‐marking process. Given that Prof. Valmed otherwise demonstrated performance largely comparable to that of non‐CE‐marked LLMs, such safety‐related restrictions may represent a clinically relevant advantage in professional medical use.

Prof. Valmed achieved results similar to those of basic commercial LLMs, when whitelisting specific professional websites (aan.com and neurology.org). As shown in a previous publication, restricting an LLM's web search to these websites can improve factual accuracy and adherence to current guidelines, while also reducing hallucinations, especially in smaller LLMs [7]. According to publicly available information at the time of writing, Prof. Valmed is based on a GPT‐4‐based architecture [19] and utilizes a large curated database comprising medical documents from a broad range of sources. However, Prof. Valmed did not demonstrate significantly higher answer quality compared with other basic LLM configurations incorporating whitelisting or RAG using a smaller number of neurology‐specific sources, suggesting no clear advantage in response quality.

### 4.2 Limitations

The method utilized in this study has previously been used to assess several other LLMs with the same neurological questions, enabling a comparison of the different tools in a neurological setting [7]. Consequently, the benchmark items and exemplary answers were publicly available prior to the evaluation of Prof. Valmed. This represents an important limitation of the present study, as potential information leakage cannot be excluded; specifically, benchmark questions or associated answer material may have been included in the model's training data. Therefore, the reported performance may partially reflect prior exposure to benchmark content rather than solely the model's reasoning capabilities. As the exact training data of Prof. Valmed are not publicly disclosed, it is not possible to determine whether prior exposure to benchmark data occurred. Future studies should validate these findings using newly developed, independent benchmark datasets that were not publicly accessible during model training.

Universally accepted guidelines on how to evaluate LLMs in the medical context are currently still lacking, which limits the comparability to other studies testing LLMs for use in different medical fields [7]. Although attempts have been made to develop standardized benchmarks applicable across different medical fields, clear frameworks for evaluating LLMs and defining performance thresholds are still needed and should ideally be supported by regulatory bodies [20, 21].

This study analyzed Prof. Valmed's answer accuracy but did not evaluate other factors, such as answer clarity, quality of reasoning or potential biases of the model. As highlighted by a recent review on medical LLM evaluations, these factors influence the usability and safety of AI tools in clinical settings [22] and should be taken into account in future studies using a more advanced benchmark.

Furthermore, ethical concerns regarding the use of LLMs in the medical field need to be discussed, for example focusing on issues such as data biases, transparency, and accountability. These issues might challenge existing conventions regarding scientific authorship and could potentially threaten patients' trust in clinical decision‐making [23]. Therefore, the development of ethical frameworks, extending beyond mere evaluations of accuracy, is essential before LLMs are widely integrated into clinical practice.

This study is further limited by the intransparency of the commercial LLM tested. The exact model and mechanisms used by Prof. Valmed are not known publicly and the reproducibility of results is limited due to possible future software updates, which might go unnoticed to the end user.

The benchmark employed in this study was established based on American guidelines, which can differ from those applicable in Europe. As both the raters in this study and the product are predominantly set in Europe, the European perspective was also considered when evaluating the provided answers. However, to keep conditions comparable, the underlying AAN guidelines served as gold standard and were in fact more often than not retrieved by Prof. Valmed. Nevertheless, a potential bias arising from the different origins of the tested LLMs and benchmark questions remains in this setup, which, however, would have similarly affected the evaluation of other LLMs using this benchmark.

### 4.3 Implications for the Future Use of LLMs in Neurology

As LLMs yield responses faster than research with traditional search engines like Google, and give better support to clinicians for information retrieval in certain scenarios, they are expected to become integral components of future clinical decision support [1, 24, 25]. Especially in the field of neurology, such tools are promising, as clear and evidence‐based guidelines, which can be integrated into LLMs through RAG or whitelisting, are available for many neurological diseases.

The role and necessity for certified medical products for information retrieval remains uncertain and yet to be explored. Prof. Valmed is the first medical LLM to receive a CE mark, paving the way for AI‐based tools to be recognized as medical products [14]. The process of obtaining a CE mark for a medical device requires substantial financial and logistical resources for testing, technical documentation, and post‐market surveillance and puts a large burden especially on small companies [26, 27]. As regulation for AI‐enabled products is still in its early development, this field is even more complex and has multiple policies in place all over Europe [28].

Furthermore, uncertainties remain regarding the possibility of updating an AI‐based product after CE‐marking, a process originally focused on certifying hardware products [29]. Significant changes to the model may require a re‐assessment of the product, potentially hindering the common process of updating currently rapidly developing models or data sources that is standard practice in the software industry [30]. This raises the question of whether a certification such as CE marking might limit the further development of digital tools such as LLMs and whether the process of certification itself might need to be rethought. One might ask what kind of medical queries that were previously typed into a Google search might warrant a certified medical product, which inevitably comes with a higher price while of course offering proper standards in data protection. Generally, the introduction of a CE‐marked product into the digital AI‐landscape represents a positive development, even though questions remain regarding which types of digital tools used in health care settings should be required to obtain such certification.

## 5 Conclusions

Prof. Valmed achieved an answer accuracy within the range of most standard commercial LLM tools on an AAN‐based neurology benchmark, with similar performance on case‐based and knowledge‐based items. It neither consistently outperformed nor underperformed its peers, while reasoning‐enabled configurations tended to achieve the highest accuracy. CE marking indicates regulatory conformity rather than inherent performance advantage. Because certification requires version stability and documented change control, CE‐marked systems may integrate new model architectures and content updates less frequently than frontier tools, creating a potential performance lag. Future work should evaluate reasoning quality, source governance, and the feasibility of timely updates within regulated products across additional clinical domains.

## Author Contributions

**Paula Z. Epping:** writing – original draft, conceptualization, investigation, writing – review and editing, data curation. **Lars Masanneck:** writing – original draft, writing – review and editing, formal analysis, investigation, conceptualization. **Sven G. Meuth:** writing – original draft, writing – review and editing, investigation, conceptualization, supervision. **Marc Pawlitzki:** writing – original draft, writing – review and editing, investigation, conceptualization, supervision. **Patricia Kirschner:** writing – original draft, writing – review and editing, conceptualization.

## Conflicts of Interest

P.K. reports no conflicts of interest related to this study. She has received honoraria for lectures, consultancy and travel support for attending meetings from Deutsche Gesellschaft für Neurologie (DGN), Hexal, Roche, Sanofi, Merck, Neuraxpharm and Viatris. P.Z.E. reports no conflicts of interest. S.G.M. reports no conflicts of interest related to this study. He received honoraria for lecturing, travel expenses and for attending meetings from Academy 2, Argenx, Alexion, Almirall, Amicus Therapeutics Germany, AstraZeneca, Bayer Health Care, Biogen, BioNtech, BMS, Celgene, Datamed, Demecan, Desitin, Diamed, Diaplan, DIU Dresden, DPmed, Gen Medicine and Healthcare products, Genzyme, Hexal AG, IGES, Impulze GmbH, Janssen Cilag, KW Medipoint, MedDay Pharmaceuticals, Medmile, Merck Serono, MICE, Mylan, Neuraxpharm, Neuropoint, Novartis, Novo Nordisk, ONO Pharma, Oxford PharmaGenesis, QuintilesIMS, Roche, Sanofi, Springer Medizin Verlag, STADA, Chugai Pharma, Teva, UCB, Viatris, Wings for Life international and Xcenda. His research is funded by the German Ministry for Education and Research (BMBF), German Federal Institute for Risk Assessment (BfR), German Research Foundation (DFG), Else Kröner Fresenius Foundation, Gemeinsamer Bundesausschuss (G‐BA), German Academic Exchange Service, Hertie Foundation, Interdisciplinary Center for Clinical Studies (IZKF) Muenster, German Foundation Neurology, Ministry of Culture and Science of the State of North Rhine‐Westphalia, The Daimler and Benz Foundation, Multiple Sclerosis Society North Rhine‐Westphalia Regional Association (dmsg), Peek & Cloppenburg Düsseldorf Foundation, Hempel Foundation for Science, Art and Welfare, German Alzheimer Society e.V. Dementia self‐help and Alexion, Almirall, Amicus Therapeutics Germany, Argenx, Bayer Vital GmbH, BGP Products Operations (Viatris Company), Biogen, BMS, Demecan, Diamed, DGM e.v., Fresenius Medical Care, Genzyme, Gesellschaft von Freunden und Förderern der Heinrich‐Heine‐Universität Düsseldorf e.V., HERZ Burgdorf, Hexal, Janssen, Merck Serono, Novartis, Novo Nordisk Pharma, ONO Pharma, Roche and Teva, all outside the scope of this study. M.P. reports no conflicts of interest related to this study. He received honoraria for lecturing and travel expenses for attending meetings from Alexion, ArgenX, Bayer Health Care, Biogen, Demecan, Hexal, Merck Serono, Neuraxpharm, Novartis, Roche, Sanofi‐Aventis, Takeda and Teva. His research is funded by ArgenX, Biogen, Hexal, Novartis, Roche, the German Multiple Sclerosis Foundation (DMSG) and B. Braun foundation, all outside the scope of this study. L.M. reports no conflicts of interest related to this study. He reports honoraria for lecturing, consulting and travel expenses for attending meetings from Biogen, Merck Serono, argenX, Roche, Alexion and Novartis, all outside the scope of this work. His research is funded by the DMSG, B. Braun foundation and the German Research Foundation (DFG)—493659010.

## Supporting information


**Table S1:** Neurological topics covered by benchmark questions.
**Table S2:** Questions Prof. Valmed refused to answer.
**Table S3:** Pairwise comparison of Prof. Valmed to other LLMs.

## Data Availability

The corresponding author will share data and the underlying code for this study to researchers who provide a methodologically sound proposal. This data can be made available following publication upon reasonable request.
