# Supplementary material for: Evaluating the First CE‐Marked LLM‐Based Chatbot for Evidence‐Based Neurology
Source: Eur J Neurol. 2026 Aug 18;33(8):e70736. doi: 10.1111/ene.70736 (PMC13484281; doi:10.1111/ene.70736)
Supplement: Supplementary file 1 [file ENE-33-e70736-s001.doc]

**Supplemental material**

**Supplemental table 1: Neurological topics covered by benchmark questions**

|  | **Neurological topic** |
| --- | --- |
| **1.** | Antiseizure medication withdrawal in seizure-free patients |
| **2.** | Teratogenesis, perinatal, and neurodevelopmental outcomes after in utero exposure to antiseizure medication |
| **3.** | Prevention, Diagnosis and Treatment of Lyme Disease |
| **4.** | Pediatric and Adult Brain Death/Death |
| **5.** | Oral and topical treatment of painful diabetic polyneuropathy |
| **6.** | Dopaminergic Therapy for Motor Symptoms in Early Parkinson Disease |
| **7.** | Pharmacologic treatment for pediatric migraine prevention |
| **8.** | Acute treatment of migraine in children and adolescents |
| **9.** | Vaccine-preventable infections and immunization in multiple sclerosis |
| **10.** | Treatment for insomnia and disrupted sleep behavior in children and adolescents with autism spectrum disorder |
| **11.** | Patent foramen ovale and secondary stroke prevention |
| **12.** | Stroke Prevention in Symptomatic Large Artery Intracranial Atherosclerosis |
| **13.** | Treatment of tics in people with Tourette syndrome and chronic tic disorders |

*The table lists the neurological topics represented in the benchmark dataset. The complete list of benchmark questions and the corresponding AAN guidelines from which they were derived are publicly available in the supplement of Masanneck L, et al. (npj Digital Medicine, 2025).*

**Supplemental table 2: Questions Prof. Valmed refused to answer**

| **Question type** | **Question entered in Prof. Valmed** |
| --- | --- |
| **Knowledge-based** | According to current AAN guidelines, what is the minimum core temperature that should be reached before a patient can be diagnosed with brain death? |
| **Case-based** | A 55-year-old male patient is admitted to the intensive care unit following a severe traumatic brain injury. He is currently showing no signs of brainstem function other than spontaneous breathing, and remains in a deeply comatose state. The intensivist on call is considering the diagnosis of brain death and needs to determine the appropriate steps. According to current guidelines, how should the physician proceed with assessing brain death in this patient? |
| **Case-based** | A 54-year-old male patient is in the ICU after a severe traumatic brain injury and is being evaluated for brain death. Clinical examination shows an absence of brainstem reflexes, and the attending physician considers using imaging as an ancillary test to confirm the diagnosis. The options include CT angiography, MRI angiography, or conventional 4-vessel catheter angiography. According to current guidelines, how should these imaging modalities be utilized to assist with the diagnosis of brain death? |

**Supplemental table 3: Pairwise comparison of Prof. Valmed to other LLMs**

| **Other Model** | **N Paired** | **Mean Difference** | **Prof. Valmed Better*** | **Other LLM Better*** | **Ties*** | **P-adj** | **P-value** | **Significant**  **difference** |
| --- | --- | --- | --- | --- | --- | --- | --- | --- |
| Mixtral-8x7b | 127 | 0·843 | 83 | 5 | 39 | P<0·001 | P<0·001 | **Yes** |
| Gemini-1.5-Pro | 127 | 0·748 | 74 | 3 | 50 | P<0·001 | P<0·001 | **Yes** |
| Llama-3.1-nemotron-70b | 127 | 0·583 | 61 | 5 | 61 | P<0·001 | P<0·001 | **Yes** |
| LLaMA3-70b-8192 | 127 | 0·575 | 64 | 4 | 59 | P<0·001 | P<0·001 | **Yes** |
| GPT-4o mini | 127 | 0·535 | 61 | 4 | 62 | P<0·001 | P<0·001 | **Yes** |
| GPT-4-Turbo | 127 | 0·457 | 53 | 7 | 67 | P<0·001 | P<0·001 | **Yes** |
| Sonar | 127 | 0·268 | 45 | 16 | 66 | P=0·014 | P=0·002 | **Yes** |
| GPT-4o | 127 | 0·197 | 32 | 9 | 86 | P=0·006 | P<0·001 | **Yes** |
| Llama-3.1-sonar-405b-online | 127 | 0·181 | 36 | 14 | 77 | P=0·144 | P=0·024 | No |
| Sonar + WL | 127 | -0·016 | 23 | 21 | 83 | P=0·903 | P=0·749 | No |
| Sonar-Pro | 127 | -0·055 | 17 | 18 | 92 | P=0·903 | P=0·301 | No |
| Sonar-Reasoning-Pro | 127 | -0·055 | 17 | 19 | 91 | P=0·903 | P=0·313 | No |
| OpenEvidence (Assumed WL) | 127 | -0·063 | 10 | 16 | 101 | P=0·769 | P=0·192 | No |
| GPT-4o +RAG | 127 | -0·118 | 12 | 20 | 95 | P=0·159 | P=0·032 | No |
| Sonar-Pro + WL | 127 | -0·126 | 8 | 18 | 101 | P=0·096 | P=0·014 | No |
| Sonar-Reasoning-Pro + WL | 127 | -0·150 | 9 | 22 | 96 | P=0·048 | P=0·006 | **Yes** |

*Wilcoxon signed-rank tests with Holm-Bonferroni correction for multiple comparisons. Positive mean differences indicate Prof. Valmed performed better; negative mean differences indicate the other model performed better. Adjusted p-values should be used for the interpretation of statistical significance; unadjusted p-values are provided for transparency. *Number of questions with higher answer accuracy in pairwise comparison.*
